# Changes and Predictors of the Sense of Meaning in Life in Polish University Students Participating in Psychological Workshops Communication–Forgiveness–Love

**DOI:** 10.1007/s10943-018-0631-1

**Published:** 2018-04-28

**Authors:** Anna Kuk, Monika Guszkowska

**Affiliations:** 1 The Faculty of Physical Education, Józef Piłsudski University of Physical Education in Warsaw, Marymoncka 34, 00-968 Warsaw, Poland; 2 The Faculty of Rehabilitation, Józef Piłsudski University of Physical Education in Warsaw, Marymocka 34, 00-968 Warsaw, Poland

**Keywords:** Sense of meaning in life, Psychological workshops, University students, Poland

## Abstract

The aim of this study was to determine changes in the sense of meaning in life of university students who participated in psychological workshops “Communication–Forgiveness–Love”. The study evaluated 33 university students from first-cycle and second-cycle studies in physical education in the Józef Piłsudski University of Physical Education in Warsaw. The Reker’s *Life Attitude Profile*-*Revised* Questionnaire, *Social Competencies Questionnaire* (*KKS*) by Matczak, *Emotional Intelligence Questionnaire* (*INTE*) by Schutte et al. and the Goldberg’s *General Health Questionnaire* GHQ-28 were used. The study found that psychological workshops can be effective in instilling the sense of meaning in life in university students, especially those from first-cycle studies. The workshops can produce more benefits to students with worse mental status and with lower social competencies.

## Introduction

For at least the last three decades, spirituality has been found to be an important or even a leading aspect of human health and wellness (Dennis and Dennis 2003; Dorsey 2000; Eberst 1984; Muller and Dennis 2007). Spirituality is understood to mean an internal sense of purpose, meaning in life (Dennis and Dennis 2003) or a positive sense of meaning and purpose in life (Adams et al. 2000). The most often mentioned indicators of spirituality include looking for and having life goals, sense of control of life and eagerness for living (Reker 2001). The sense of meaning in life relates to the process of discovering of the importance of living (Vela et al. 2015). This sense has been conceptualized as “having” the sense of meaning in life (attributing meaning to your own life) and looking for the meaning in life (motivation for finding its meaning) (Steger and Shin 2010).

The sense of meaning in life allows for predicting the intensity of the symptoms of depression and anxiety (Vehling et al. 2011), thus being related with mental wellness. It is positively correlated with the sense of happiness (Vela et al. 2016). Its effect on the perceived wellness is mediated by personal psychological resources, such as optimism or a sense of coherence (Adams et al. 2000).

Sense of the meaning in life increases from the late period of adolescence to mid-adulthood (Reker et al. 1987). More in-depth analysis suggested that as a person ages, death acceptance and having your own goal in life may increase, whereas looking for new goals decreases (Reker et al. 2003). Furthermore, Gupta (2016) documented the highest level of the sense of meaning in life and satisfaction from life in people aged 40–58 years. It was significantly higher compared to people aged 25–29 years and those over 64. However, these differences have not always been found (Halama et al. 2014; Steger et al. 2009).

The end of adolescence followed by the onset of early adulthood represents one of the transition periods in human life. Life patterns are established during studying at university, having effect on the future stages of life. In this period, young people look for their own place in life and experiment with various roles which they would like to play. They also experience a number of important life-changing events, which impact on their spirituality (King et al. 2001; Muller and Dennis 2007). As demonstrated in the study by Muller and Dennis (2007), a plethora of changes in life are linked to a stronger sense of existential emptiness and more eager looking for new goals. Among young people, the sense of meaning in life is negatively correlated with the perceived stress (Halama et al. 2014). The effect of stress on sense of meaning in life is mediated by personality traits. These moderators vary depending on age. In young adults, these functions are performed by extroversion and agreeableness (Halama et al. 2014).

Sex can be a differentiating factor in the sense of meaning in life. Presence of intersexual differences was suggested by the analyses of Reker (2005). Dennis, Muller and Miller (2004) found the strongest sense of meaning in life in American female college students compared to their male peers. Their goals were more transparent, while the sense of being here was stronger. The results obtained for men reflected lower life satisfaction and looking for identity.

The period of study at university is marked by significant life transitions which are likely to impact on students’ spirituality. Spirituality is a leading component of mental health and remains in close relation to its other dimensions, thus affecting perceived wellness (Adams et al. 2000). It seems to be determined by personality (Halama et al. 2014) and possible to be formed (Muller and Dennis 2007). For these reasons, numerous health psychologists and health educators have emphasized the need for taking actions to instil the sense of meaning in life in their students (Adams et al. 2000; Dennis and Dennis 2003; Muller and Dennis 2007). This can be achieved by, for example, dedicated student’s books used to explore their own spirituality (Atkinson 2001). It is also important to develop personal resources such as optimism and sense of coherence (Adams et al. 2000) and social competencies (e.g. assertiveness, communication skills, problem-solving skills and coping strategies). Vela, Lenz, Sparrow and Gonzalez (2016) emphasized the opportunities to use Victor Frankl’s logotherapy (existential therapy) (1963) and narrative therapy (White and Epson 1990) to increase the sense of meaning in life. It also seems possible to use psychological workshops for the purpose. Their effectiveness was demonstrated by, for example, Vela et al. (2015).

Workshops are a very intensive form of learning, with their essence consisting in gaining new experiences through activity in a group. Interactions between participants help them recognize their own abilities and those of other participants, and use them effectively. During the workshops, the participants develop cooperation, goal setting and problem-solving skills. The classes are held in the atmosphere of mutual respect, according to the specific rules and with discretion (Vopel 2000).

The importance of such measures to prevention of mental disorders and health promotion was discussed by Layous et al. (2014). Positive effects in the form of the improvements in positive mental health indices (sense of happiness, life satisfaction, positive affect, self-esteem, self-efficacy, optimism) and decreased mental problem indices (depression, anxiety) were observed in adults, adolescents and children (Odou and Vella-Brodrick 2013; Roth et al. 2017; Senf and Liau 2013; Shoshani and Steinmetz 2014; Suldo et al. 2015).

In 2002, Irish students of nursing participated for the first time in a learning module that combined psychology lectures with practical workshops on interpersonal communication. According to the students, the classes would help them work with patients after completion of the course (McCarthy et al. 2014). The findings of a study of Hongkong students pointed to the opportunities for mental health promotion through emotional intelligence training and social problem-solving skills training (Chow et al. 2011). The effectiveness of psychological workshops in the Polish academic community and involvement of students in the program were supported by our previous findings (Kuk et al. 2015). Social competencies and emotional intelligence of students who participated in interactive classes were substantially higher following 32-h workshops.

The basic question was whether the sense of meaning in life could be modified through intended effects by means of the methods used in psychological workshops. The aim of this study was to determine changes in the sense of meaning in life of university students who participated in psychological workshops “Communication–Forgiveness–Love”.

While taking actions oriented at the development of specific mental properties, the question arises whether there are factors that could determine their effectiveness. Therefore, the study also attempted to establish what the predictors of these changes are. They were expected to be personal factors (age, sex and university course level), social competencies and mental status of the participants.

## Material and Method

### Study Participants

The study examined 33 university students (including 15 men and 18 women) of the first-cycle (*n* = 18) and second-cycle (*n* = 15) studies in physical education from the Józef Piłsudski University of Physical Education in Warsaw aged 19–35 years (*M* = 21.91; SD = 3.521). The participants from the first-cycle studies were significantly younger than those from the second-cycle studies (by 19.78 ± .878 and 24.47 ± 3.815, respectively, *p* < .001). The participants volunteered to take part in psychological workshops on problems of communication, forgiveness and love that were not included in academic curricula. The workshops were organized as extracurricular activities.

## Method

The study was organized within a quasi-experimental design that evaluated pretest–posttest values of the dependent variable (sense of meaning in life). The intervention was aimed to improve the ability to control emotional states, modify cognitive patterns and expand the array of communicational behaviours in students who are expected to become physical education teachers and coaches. The intervention was organized in the form of workshops prepared by psychologists who are employees of the Józef Piłsudski University of Physical Education in Warsaw. The first aim of the workshop “Communication in light of experienced feelings and emotions” was to identify intrapsychic processes, such as emotions, feelings and cognitive patterns which underlie the interpersonal communication. The students learnt active listening skills, using the “Me” messages, replacement of “You” messages into “Me” messages, overcoming barriers to communication, reading non-verbal messages, distinguishing between submissive and negative or aggressive behaviours while assertive behaviours were instilled. During the forgiveness part of the workshops, a model used to identify and solve conflict situations was presented. Students recognized their own coping strategies and familiarized with the method of six steps in problem solving. The phases of the forgiveness process were analysed with respect to their own experiences and using the examples from literature and films. The third workshop, The Need for Love, was devoted to the process of love development into mature love. The participants learnt different types of love and various languages to express feelings. They made self-assessment of their attitudes towards love and love language. The problems of emotional co-dependency and building a constructive self-image and self-esteem were also discussed.

The workshops were divided into three weekend blocks, separately for the students from first-cycle and second-cycle studies. The classes in both groups were designed according to the same scenario and schedule. Each workshop was held on Saturdays and Sundays for 12 h in total, with total duration of the workshops of 36 hours.

The assessment of the sense of meaning in life was performed using the *Life Attitude Profile*-*Revised* (*LAP*-R) by Reker (2001) with Polish adaptation by Klamut (2010). This questionnaire is composed of 48 statements evaluated on a scale of 1 (*I strongly agree*) to 7 (*I strongly disagree*). It contains the scales concerning the purpose, coherence, life control, death acceptance, existential vacuum and goal seeking. The results are computed by summing the scores. Furthermore, the personal meaning index (sum of the scores obtained from the two dimensions: purpose and coherence) and balance of attitudes towards life—existential transcendence (sum of scores on the dimension of existential vacuum and goal seeking subtracted from the sum of the scores on all simple scales) can be derived. The tool is characterized by satisfactory internal consistency and internal stability indices. The reliability indices range from 0.80 to 0.84. The validity of the questionnaire was supported by the results of factor analysis and correlations with two other tests that measured the sense of meaning in life based on existential psychology, which substantially supported the adopted structure (Klamut 2010). The *LAP*-*R* sessions were performed in groups, immediately before the first workshop and following the last workshop (6 weeks later).

Furthermore, other inventories were used: *The Social Competencies Questionnaire* (*KKS*) by Matczak (2001), *The Emotional Intelligence Questionnaire* (*INTE*) by N.S. Schutte et al. adapted by A. Jaworowska and A. Matczak (2001) and *The General Health Questionnaire* (*GHQ*-*28*) by Goldberg (2001).

The *KKS* questionnaire developed by Matczak (2001) is composed of 90 statements, including 90 diagnostic and 30 non-diagnostic items. The respondent evaluates the effectiveness of each activity on a scale from 1 to 4. Three dimensions are used: competencies that determine the effectiveness of behaviours in intimate situations, during social exposure and those that require being assertive. A total index of social competencies is also computed. The tool is characterized by satisfactory reliability and validity. Cronbach’s alpha in the group of university students ranges from 0.76 to 0.89, whereas internal stability indices range from 0.71 to 0.85.

The *INTE* questionnaire (Jaworowska and Matczak 2001) comprises 33 statements responded by choosing one of five answers (from 1—*I strongly disagree* to 5—*I strongly agree*). The tool is recommended due to its high reliability (Cronbach’s alpha for the group of university students is 0.84), both internal consistency and result stability. The results of factor analyses demonstrated that the test structure is consistent with the theoretical model of emotional intelligence by Salovey and Meyer (1990).

*The General Health Questionnaire* (*GHQ*-*28*) by Goldberg (Goldberg and Williams 2001) was used to evaluate mental status. It is composed of 28 items in 4 subscales: somatic symptoms, anxiety and insomnia, social dysfunction and depression symptoms. A total score can also be computed. The tool is characterized by high internal consistency and satisfactory absolute stability. Cronbach’s alpha obtained from various studies ranged from 0.82 to 0.93. Test validity was confirmed using the intergroup comparison method and factor analyses of the *GHQ*-*28* version.

All participants gave their written consent to participate in the research. The research project was approved by the Senate’s Research Bioethics Commission, Józef Piłsudski University of Physical Education in Warsaw. The research was conducted in accordance with the Declaration of Helsinki on human research.

In order to establish the differences between the measurements of the sense of meaning in life, we used the nonparametric Wilcoxon signed-rank test. Furthermore, the stepwise regression analysis was employed. The statistical significance of the results was set at *p* = .05.

## Results

Comparison of the pretest and posttest results for the sense of meaning in life (Table 1) reveals significant changes in four of six simple subscales and in both complex subscales. After completion of the workshops, participants (university students) demonstrated stronger confidence in having the purpose that determines their meaning in life (purpose), stronger sense of order in life and self-identity (coherence). Their confidence in their ability to change their own lives (choice) became more established. The experiencing of boredom, apathy and no purpose and goals in life (existential vacuum) was reduced. Level of death anxiety and readiness to accept death as a natural end of life (death acceptance) and the need for abandoning the routines of life and searching for new experiences (goal seeking) remained unchanged. A significant increase was also found for the sense of meaning in life, which includes having important life goals and coherent self-understanding, understanding others and life in general (personal meaning index), life satisfaction and level of its acceptance (existential transcendence).

**Table 1:** Changes in the meaning in life of workshop participants (*n* = 33)

Measurement variable	Before the workshops *M* ± SD	After the workshops *M* ± SD	Wilcoxon test	
			*Z*	*p*
Purpose	39.58 ± 6.78	43.06 ± 6.63	− 2.746	.006
Coherence	39.97 ± 6.30	43.21 ± 5.15	− 3.014	.003
Choice	44.30 ± 5.73	48.00 ± 5.11	− 3.228	.001
Death acceptance	35.88 ± 10.49	37.39 ± 9.86	− 1.142	.254
Existential vacuum	27.21 ± 7.48	24.18 ± 6.62	− 2.659	.008
Goal seeking	41.76 ± 5.40	42.45 ± 7.28	− .565	.572
Personal meaning index	79.55 ± 12.07	86.27 ± 11.24	− 3.192	.001
Existential transcendence	90.76 ± 28.41	105.15 ± 25.84	− 3.065	.002

Signs: *M* arithmetic average, *SD* standard deviation, *Z* result of the Wilcoxon signed-rank test, *p* level of significance

Table 2 presents the results of the last step of the stepwise regression analysis. The dependent variables were indices of change in each dimension of the sense of meaning in life computed by subtracting of the first from the second measurement. They were established only for the simple and complex subscales where significant changes were observed (see Table 1). The factors were personal variables (sex, age, level of university studies), indices of social competencies, emotional intelligence and psychological difficulties (somatic symptoms, anxiety and insomnia, social dysfunction, depression symptoms) before workshops.

**Table 2:** Predictors of changes in the sense of meaning in life in workshop participants

Dependent variable	Predictor	Beta	Model (*R*^2^, *F*, *p*)
Increase in purpose	Level of university studies	− .380	.117; 5.230; .029
Increase in coherence	Depression	.385	.258; 6.565; .004
	Level of university studies	− .356	
Increase in choice	Level of university studies	− .394	.344; 9.382; .001
	Competencies in the situations of social exposures	− .356	
Decline in existential vacuum	Depression	− .635	.384; 20.957; < .001
Increase in personal meaning index	Level of university studies	− .377	.261; 6.650; .004
	Depression	.368	
Increase in existential transcendence	Level of university studies	− .443	.520; 9.665; < .001
	Depression	.429	
	Emotional intelligence	.435	
	Competencies in the situations of social exposures	− .298	

The especially important predictors of changes in the sense of meaning in life were the level of university studies and intensification of depression symptoms. The first factor is a negative predictor of changes, which means that bigger changes can be expected in students of the first-cycle studies. In this group of students, more pronounced improvements in all positive dimensions of the sense of meaning in life can be expected, i.e. purpose, coherence, choice and the indices of personal meaning and existential transcendence (scores on complex scales).

The other important predictor is depression level. A greater increase in coherence, sense of personal meaning, balance in attitudes towards life (existential transcendence) and lower existential vacuum can be expected in students with greater intensity of depression symptoms before the workshops.

Social competencies in the situations of social exposure are a negative predictor of changes in the sense of life control (choice) and balance of attitudes towards life (existential transcendence). Greater increase can be expected in students with lower competencies in situations of social exposure revealed before the workshops.

In the case of changes in the existential transcendence, emotional intelligence turned out to be also critical, being its positive predictor.

The factors taken into consideration in the study ensure the best (52%) prediction of changes in the results in the subscale of existential transcendence, which provides the general index of attitudes towards life: degree of experiencing of the meaning in life and motivation for seeking the purpose. It can be considered an index of general life satisfaction and life acceptance. A substantial increase in life satisfaction can be expected in students from first-cycle studies, who revealed greater intensity of depression symptoms before the workshops, were more intelligent emotionally and had better competencies in situations of social exposure.

Coefficients of determination are also relatively high in the case of the decline in existential vacuum: it can be predicted in over 38% of the cases, only based on the index of depression intensity before the workshops. Coefficient of determination had similar value for the increase in choice (life control). This change can be predicted in over 34% based on the level of studies and social competencies in the situation of exposure. Greater intensification of the confidence in life control should be expected in students from first-cycle studies and those with low social competencies in the situations of public performance.

## Discussion

The study found a significant increase in the sense of meaning in life in four basic dimensions (increase in the choice, coherence, purpose and lower existential vacuum) and increase in values of complex indices of personal meaning and existential transcendence in participants of psychological workshops devoted to the problems of communication, forgiveness and love. These results suggest a positive answer to the basic research question whether the sense of meaning in life of a person can be formed by providing him or her with the experiences during interventions in the form of psychological workshops. This is consistent with the effectiveness of such interventions predicted by other authors (Vela et al. 2015, 2016). Since the contents of the workshops were related in a sense to the assumptions of the Frankl’s logotherapy, the study confirms the usefulness of this approach in the development of the sense of meaning in life. This was suggested in a previous study by Vela et al. (2015, 2016).

However, as the study was organized within a single group design, it cannot be excluded that the changes were not linked to the intervention. They can represent a manifestation of a spiritual development of the students which is unrelated to participation in the workshops. However, it seems to be little probable that such critical changes in the sense of meaning in life occurred spontaneously in the period of nearly 2 months. The research based on the experimental model with the experimental and control groups would at least partially exclude these doubts. According to Steger et al. (2012), the lead role in the development of the sense of meaning in life during adolescence is played by social-cognitive factors, such as cognitive development, identity, self-understanding and social modelling. It stems from the ability to exist in the social world, self-regulation and autonomy.

It is worth noticing that before the workshops, the participants were characterized by the level of the sense of meaning in life which was slightly higher than average normal levels determined by the Polish population (Klamut 2010). On two simple scales (purpose and coherence) and both complex scales, over half of the respondents obtained the scores higher than the sixth sten. On the scale of existential vacuum, two-thirds of students had scores below the fifth sten. Despite this fact, a significant increase in point indices of the attitudes from the positive aspect and a decline in the index of existential vacuum were documented. The increase in standardized (sten) scores was found mostly (in ca. 60% of the participants) on the scale of cohesion and choice and on both complex scales. On other scales, the increase occurred in around half of the participants. Similar pattern was observed in the case of the decline in sten scores on the scale of existential vacuum. Further analyses are needed to determine the degree of dependence of these changes on the initial level of the sense of meaning in life.

It should be noted that the participants were volunteers, who had known the contents of activities before the workshops began. Therefore, the level of the sense of meaning in life recorded for the first examination can differ from the level that could have been found in those who did not want to participate in the workshops. Meaning in life can adopt various places in the hierarchy of values of a person (Klamut 2002). It is likely that the university students who volunteered to participate in the workshops were more motivated to look for their meaning in life and had more or less satisfied spiritual needs.

The aim of the study was also to determine the factors connected with changes in the components of the sense of meaning in life and their predictors. Level of university studies was a significant negative predictor of the increase in the level of the three existential attitudes (positive aspect) and the scores on both complex scales. Therefore, greater increase in the sense of meaning in life can be expected in students from first-cycle studies. Obviously, the level of education is related to age, with students from master’s courses being significantly older than people from first-cycle studies. Age differentiates the sense of meaning in life. Results of previous studies (Gupta 2016; Reker et al. 1987, 2003) suggested that particularly significant changes are observed in the sense of meaning in life in the transition period from late adolescence to early adulthood. However, age was not significantly correlated with changes in the sense of meaning in life and was not a predictor of these changes. Therefore, it can be reasonably presumed that it is the scope of life experiences that matters rather than age. According to Fry (1998), in order to acquire the sense of meaning in life, the adolescents have to develop psychological resources (ability to effectively cope with social expectations) and social resources (support from family and peers). Self-efficacy and self-regulation abilities can also be important (Brassai et al. 2013).

As demonstrated by the results of the analyses not presented in this study, university students from both groups also differed statistically significantly in terms of the initial sense of meaning in life (apart from the choice and goal seeking) Stronger sense of meaning in life was found in the university students from second-cycle studies, which is consistent with the assumption of the importance of life experiences. After the workshops, they were characterized by only lower level of existential vacuum compared to the students from first-cycle studies. Interestingly, the latter revealed a slightly higher level of the sense of life control (choice) in the second examination, with the difference reaching the level of tendency. This shows that interventions aimed at the development of the sense of meaning in life can be more effective in the case of people with lower level of life control. It remains unclear whether this is caused by a simple ceiling effect: it is difficult to improve something which is already at a high level. This can be also affected by the scope and character of life experiences. Undoubtedly, these questions need to be further explored.

It is worth noting the division into “having” the sense of meaning in life (attributing meaning to your own life) and looking for the meaning in life (motivation for finding its meaning) (Steger and Shin 2010). Having the sense of meaning in life is positively correlated with mental health (Brassai et al. 2011, Steger and Shin 2010; Vela et al. 2014), self-esteem (To et al. 2014), perceived competencies (Utvaer 2014) and optimism (Steger and Frazier 2005). Students with strong sense of meaning in life are more successful in the achievement of goals and more willing to find positive aspects of negative situations (Dogra et al. 2011). On the other hand, looking for the meaning in life can be linked to no hope in the opportunities for the achievement of goals (Vela et al. 2014). It is likely that the use of the tool that features such a differentiation (Steger and Shin 2010) would provide additional insights into the factors that determine workshop effectiveness.

Depression turned out to be an important positive predictor of the increase in the sense of coherence and decline in the sense of existential vacuum and the increase in both complex indices. Therefore, the findings suggest that the workshops can yield benefits to students with worse mental status. Consequently, on the one hand, the sense of meaning in life can be a predictor of intensification of depression symptoms (Vehling et al. 2011), while on the other hand, depression allows for predicting changes in the sense of meaning in life in participants of the psychological workshops. This is consistent with the findings of other authors who demonstrated correlations between the sense of meaning in life and intensity of depression symptoms (Katsogiann and Kleftaras 2015; Wilchek-Aviad and Malka 2016).

Similar pattern is observed in the case of social competencies. Greater increase in the sense of meaning in life can be expected in university students with lower competencies in the situation of social exposure. As argued by Fry (1998), this is one of the resources which determines the development of the sense of meaning in life. People with poor abilities to cope with social expectations can develop them more during the workshops. This is likely to lead to the greater increase in the sense of meaning in life.

Care for the sense of meaning in life should represent one of the measures in mental health promotion programs. Katsogiann and Kleftaras (2015) emphasized the importance of the development of spirituality and sense of meaning in life in addiction treatment, and prevention and treatment of depression of addicted people. The usefulness of implementation of programs for the development of the sense of meaning in life in suicide prevention was evaluated in a study of Jewish young people conducted by Wilchek-Aviad and Malka (2016). These researchers found a significant negative correlation between the sense of meaning in life and suicidal tendencies and between the sense of meaning in life and depression, sadness, anxiety and feeling of guilt and anger. Therefore, the lack of the sense of meaning in life is reflected by the intensification of depression symptoms. Over 300 million people all over the world suffer from depression (WHO 2017). In the extreme cases, depression can lead to suicide attempts. Nearly 800,000 people every year die following suicide attempts. Among young people aged 15–29 years, suicide is the second most prevalent cause of deaths. Also for these reasons, initiatives oriented at the development of spirituality are critical.

Our study has some limitations. The first limitation is one-group study design, which does not allow for cause-and-effect reasoning. With no control group, it is impossible to distinguish between the effects of intervention from changes occurring spontaneously in time or caused by uncontrollable factors. The other limitation is small size of study groups that limited opportunities for generalization of the results. Furthermore, the study did not take into consideration the previously established determinants of the sense of meaning in life (at least personality traits) which could have affected the participants of the psychological workshops. Despite these limitations, the results obtained in our study suggest that the psychological workshops can be effective in instilling the sense of meaning in life in university students, especially those from first-cycle studies. The workshops can produce more benefits to students with worse mental status and with lower social competencies.
